# Immune Dysregulation in Bipolar Disorder: The Role of IL-27 and EBI3

**DOI:** 10.1192/bjo.2025.10182

**Published:** 2025-06-20

**Authors:** Golnaz Sheykhnazari, Maliheh Ziaee, Ezinne Ijeoma Onuba, Najmeh Shahini, Yaser Bagheri

**Affiliations:** ^1^Golestan Research Center of Psychiatry (GRCP), Golestan University of Medical Sciences, Gorgan, Iran, Islamic Republic of; ^2^Department of Community Medicine, School of Medicine, Social Determinants of Health Research Center, Gonabad University of Medical Sciences, Gonabad, Iran, Islamic Republic of; ^3^Erdington & Kingstanding CMHT, Birmingham and Solihull Mental Health NHS Foundation Trust, Birmingham, United Kingdom; ^4^Erdington & Kingstanding CMHT, Birmingham and Solihull Mental Health NHS Foundation Trust, Birmingham, United Kingdom; ^5^Leicestershire Partnership NHS Trust, Leicester, United Kingdom; ^6^Clinical Research Development Unit (CRDU), Agh ghala Hospital, Golestan University of Medical Sciences, Gorgan, Iran, Islamic Republic of; ^7^Clinical Research Development Unit (CRDU), Agh ghala Hospital,Golestan University of Medical Sciences, Gorgan, Iran, Islamic Republic of; ^8^Immunology Department, Faculty of Medicine, Golestan University of Medical Sciences, Gorgan, Iran, Islamic Republic of

## Abstract

**Aims:** Bipolar disorder (BD) is a severe psychiatric illness characterized by alternating depressive and manic episodes. The exact cause of BD remains unclear, but inflammatory and immunological processes are believed to play a significant role in its pathophysiology. Immune system dysregulation is a key factor, with pro-inflammatory markers like CRP, TNF-α, and IL-6 being elevated during mood episodes. Interleukin 27 (IL-27), which has both pro-inflammatory and anti-inflammatory properties, has not been extensively studied in BD. This study aims to investigate the levels of IL-27 and its subunit EBI3 in BD patients to better understand their role in the disease.

**Methods:** A cross-sectional study was conducted at Panj-Azar Hospital in Gorgan, Iran, from March 2023 to August 2023. The study included 75 patients with bipolar disorder (depression, mania) and 30 healthy controls. Participants were aged 18–65, diagnosed with bipolar disorder by two psychiatrists using DSM–V criteria, and undergoing treatment with atypical antipsychotics. Exclusion criteria included other psychiatric illnesses, substance use disorder, corticosteroid use, autoimmune/inflammatory diseases, pregnancy/breastfeeding, chronic schizophrenia, and other mental diseases. Blood samples were collected and stored at −80°C, and serum levels of IL-27 and EBI3 were measured using high-sensitivity ELISA kits. Descriptive and inferential statistics were applied to analyse the data, including normality tests, one-sample t-tests, independent t-tests, and Pearson correlation. The study followed the STROBE checklist to ensure high-quality reporting of observational studies.

**Ethical consideration**: This study was conducted after obtaining ethical approval (IR.GOUMS.REC.1400.010) from the Golestan University of Medical Sciences. Written and oral informed consent was obtained from patients

**Results:** The study revealed significant changes in the immune system of bipolar disorder (BD) patients, with IL-27 levels showing a notable difference between BD and control groups (p≤0.05). IL-27, which has dual roles in inflammatory reactions, correlated positively with ALP (p=0.05, r=−0.22), FT4 (p=0.01, r=−0.29), and CPK (p=0.03, r=−0.24), and negatively with disease duration (p=0.03, r=−0.26), suggesting its potential as a therapeutic target. EBI3 did not show significant correlations with any variables (p≥0.05).

**Conclusion:** This study highlights the significant role of immune system dysregulation in bipolar disorder (BD), particularly the elevated levels of IL-27 in BD patients compared with controls. The correlations between IL-27 and various clinical parameters suggest its potential as a biomarker and therapeutic target. Although EBI3 did not show significant correlations, the findings underscore the importance of inflammatory and immune markers in understanding BD’s pathophysiology. Further research is needed to confirm these results and explore the underlying mechanisms, which could lead to the development of new diagnostic and treatment strategies for BD

